# Alternating electric fields can improve chemotherapy treatment efficacy in blood cancer cell U937 (non-adherent cells)

**DOI:** 10.1186/s12885-023-11339-7

**Published:** 2023-09-12

**Authors:** Elham Homami, Bahram Goliaei, Seyed Peyman Shariatpanahi, Zahra Habibi-Kelishomi

**Affiliations:** Institute of Biochemistry and Biophysics, University of Tehran, PO Box 13145-1384, Tehran, Iran

**Keywords:** AML, Electric field, Daunorubicin, Combination therapy, Membrane permeability

## Abstract

**Background:**

Recent achievements in cancer therapy are the use of alternating electrical fields at intermediate frequencies (100–300 kHz) and low intensities (1–3 V/cm), which specifically target cell proliferation while affecting different cellular activities depending on the frequency used.

**Methods:**

In this article, we examine the effect of electric fields on spherical suspended cells and propose the combination of Daunorubicin, a chemotherapy agent widely used in the treatment of acute myeloid leukemia, with electric field exposure. U937 cells were subjected to an electric field with a frequency of 200 kHz and an intensity of 0.75 V/cm, or to a combination of Daunorubicin and electric field exposure, resulting in a significant reduction in cell proliferation. Furthermore, the application of an electric field to U937 cells increased Daunorubicin uptake.

**Results:**

Apoptosis and DNA damage were induced by the electric field or in conjunction with Daunorubicin. Notably, normal cells exposed to an electric field did not show significant damage, indicating a selective effect on dividing cancer cells (U937). Moreover, the electric field affects the U937 cell line either alone or in combination with Daunorubicin. This effect may be due to increased membrane permeability.

**Conclusions:**

Our findings suggest that the use of electric fields at intermediate frequencies and low intensities, either alone or in combination with Daunorubicin, has potential as a selective anti-cancer therapy for dividing cancer cells, particularly in the treatment of acute myeloid leukemia. Further research is needed to fully understand the underlying mechanisms and to optimize the use of this therapy.

## Background

AML (acute myeloid leukemia) is a common form of leukemia in adults, with approximately 1 million new cases and 147,100 deaths worldwide each year. The first-line treatment for AML involves intensive chemotherapy using drugs like daunorubicin [1]. DNR is an anthracycline antibiotic commonly used in the treatment of acute myeloid leukemia [2], and is known to induce apoptosis and DNA double-strand breaks in myeloid leukemic cells [3]. However, resistance to these drugs can develop over time, leading to relapse and poor outcomes. Higher doses or longer durations of chemotherapy may be necessary to treat resistant leukemic cells, but this can result in harmful side effects [1]. Today, scientists are looking for ways to increase the effect of chemotherapy, so combined treatments such as radiation therapy, hyperthermia, etc. with chemotherapy. In this research, we employed TTFS as a complementary approach alongside chemotherapy to enhance the efficacy of chemotherapy drugs. This choice was motivated by the fact that TTFS has been approved by the FDA (Food and Drug Administration) as a side-effect-free method for treating cancer [4]. Based on these findings, our research aims to investigate the influence of electric fields on leukemia cells(U937) in combination with the chemotherapy drug DNR.

A recent advancement in cancer therapy has been the application of alternating electrical field with intermediate frequencies (100–300 kHz) and low intensities (1–3 V/cm), as a new method for cancer treatment. TTFields (Tumor Treating Fields) have been shown to be an effective treatment for solid tumors in vitro and in vivo [5]. Moreover, TTFields are mainly effective in dividing cells; while there was no significant difference in quiescent cells comparatively [6]. TTFields are proposed to have different mechanisms of action. One of them is to interfere with metaphase, by preventing the formation of mitotic spindle; the field lines align the polar tubulins and disrupt the polymerization of microtubules. Another mechanism is related to dielectrophoresis (DEP) [7, 8], which is a phenomenon where the electric field can move polar organelles and macromolecules. This happens in late cytokinesis, where the field density of TTFields is higher, and the electric field moves these polar entities to the furrow of newly formed daughter cells [8, 9]. These mechanisms are not without critics; they claim that the orientation of tubulin dimers [9], is not affected by TTFields, because the thermal energies from Brownian motion are much higher than the dipole energy from TTFields. Also, the DEP forces from the alternating electrical field can move polar particles during telophase, but the movement is too slow due to the viscous forces. Therefore, telophase is not significantly affected by the DEP forces [10]. Some studies suggest that the cell membrane potential, which is vital for many cellular functions and processes, can be altered by an electrical field. This may affect how cells divide by changing the balance of ions inside and outside the cell. The effects of the electrical field, on tumor cell membrane potential, may provide an explanation for experiments that have observed abnormal spindle structure as well as abnormal prolongation of early mitosis during prophase [10].

According to research, the regulation of microtubule polymerization in cells is influenced by the presence of Ca++. Since the concentration of extracellular Ca++ is higher than that of intracellular Ca++, opening the Ca++ ion channel due to induced membrane voltage can lead to a decrease in microtubule polymerization. Recent experiments investigating changes in Ca++ concentration under TTFields have shown an increase in cytoplasmic Ca++ [11]. These findings indicate that the effects of the electric field on cells are not limited to mitosis, as previously believed.

The shape of a cell can affect the way that the electric field’s intensity is distributed, with non-uniform intensity being more prevalent in irregularly-shaped cells than in spherical ones [12]. Given that blood cells are typically spherical; it is pertinent to determine whether electric fields have comparable effects on non-adherent spherical cells. To address this question, a study was conducted to explore the impacts of electric fields on spherical and non-adherent blood cancer cells. The findings of this study could provide valuable insights into the potential effects of electric fields on cells in the bloodstream.

Previous research has suggested that combining electric fields with drugs [13–20] like paclitaxel or doxorubicin can lead to improved therapeutic outcomes by reducing the proliferation and viability of solid tumor cells [21]. In our study, we investigated the effectiveness of combining Daunorubicin (DNR) with electric fields. DNR is an anthracycline antibiotic commonly used in the treatment of acute myeloid leukemia [2], and is known to induce apoptosis and DNA double-strand breaks in myeloid leukemic cells [3].

## Materials and methods

Our study focused on assessing the potential synergistic effects of electric fields on the U937 cell line, a model for human acute myeloid leukemia, both alone and in combination with DNR chemotherapy treatment. The aim of this study is to provide evidence for the potential benefits of using electric fields in combination with chemotherapy for treating AML.

### Culture conditions

The monocytic U937 cell line, obtained from the National Cell Bank of Iran (Pasteur Institute, Iran), was cultured in RPMI-1640 medium (Gibco) supplemented with 10% fetal bovine serum (Gibco). Human B Lymphoblastoid Fs-204 cells, used as a normal cell line (EBV(Epstein-Barr virus) transduced), were purchased from the Iranian Biological Resource Center (IBRC) [22], and cultured under the same conditions but with 20% fetal bovine serum. Both cell lines were maintained in a 37 ˚C incubator with a humidified atmosphere containing 5% CO2. Daunorubicin (DNR) was purchased from CIPLA LTD in India, dissolved in sterile deionized water, and stored at 4 ˚C.

### Experimental setup for electric field

#### The electrodes

**Fig. 1:**
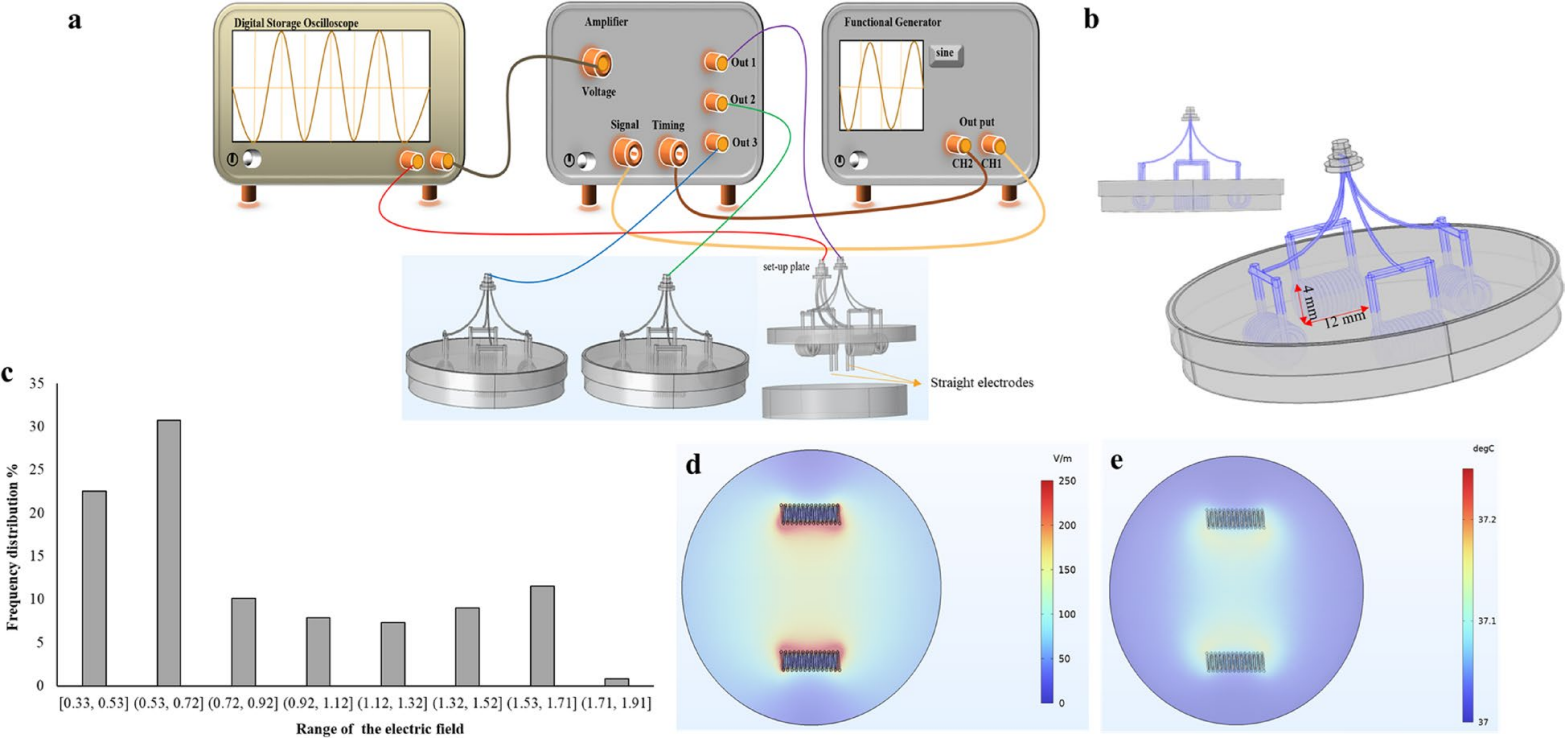
A schematic diagram of the experimental setup used to simulate the distribution of electric fields and temperature in a petri dish, using Comsol software. **a** The electric field production set-up includes; Generator (to generate the electric field), an amplifier (to amplify the intensity of the generated electric field), an oscilloscope (to monitor the magnitude of the generated electric field), and three petri-dishes (one of them were connected to the oscilloscope as a set-up petri-dishes). **b** To conduct the experiment, four electrodes used. These electrodes were affixed to the underside of the plate, while their outer surfaces were not submerged in the growth medium. Each electrode was 12 mm in length and 4 mm in height and had 14 turns of wire winding. **c** Comsol Multiphysics simulation shows the percentage distribution of the electric field in the petri dish. **d** The Distribution of the electric field changes in the petri dish was shown in the range of 0 to 250 v/m. **e** The distribution of the temperature changes in the petri dish was shown in the range of 37 to 37.2 ˚C

The experimental setup consisted of four electrodes made of copper lacquered wire, which were arranged in a glass petri dish to apply a low-intensity alternating electric field to the cells. The biocompatibility of the wire was tested to ensure it would not have any adverse effects on the cells. The lacquered wire was used to prevent electrolysis or ion release on the surface of the metallic electrodes. Two sets of double electrodes were used to apply the electric field in both the x and y directions, (Fig. 1a) which were generated sequentially by switching the output of the amplifier between two pairs of electrodes every 2 s [23]. To expose the cells to the electric field, the electrodes were connected to a sinusoidal function generator and an amplifier (Partofarazane Farda, Iran). The passing current had an intensity of approximately one milliampere, and the lacquered wire and cell culture medium formed a 10pF capacitor.

The copper ion concentration was investigated using Inductively Coupled Plasma-Optical Emission Spectroscopy (ICP-OES) [24], and the results showed an average concentration of 0.3 ppm in the sham exposure sample and 0.6 ppm in the sample under the electric field after 48 h of exposure, which was not statistically significant. The maximum allowed contaminant level targeted for Cu is 1.3 ppm, which has been set by the United States Environmental Protection Agency [25]. Additionally, the average pH of the sham sample and the sample under the electric field showed no significant difference, with values of 7.43 and 7.41, respectively. These findings suggest that the experimental setup was safe and did not cause any significant contamination or pH changes that could affect the cells.

#### The applied electric field

A variable voltage was applied to the cells using a signal generator connected to an amplifier, which consisted of a self-inductance and capacitor bank to apply the electric field while resonating with the formed capacitors in the petri dish. The intensity and frequency of the electric field were adjusted to be between 0.25–1.5 V/cm and 100–300 kHz, respectively, and were applied for 24, 48, and 72 h. The electric field’s intensity and frequency were monitored using straight electrodes temporarily positioned in the culture and attached to an oscilloscope (Fig. 1a). We utilized a total of four electrodes, which were arranged in pairs and positioned opposite each other within the plate. These electrodes were affixed to the underside of the plate, with only their outer surfaces not submerged in the growth medium. Each electrode measured 12 mm in length and 4 mm in height. Additionally, each electrode was equipped with 14 turns of wire winding. Of course, the entire electrode has the same electrical potential, and there is no current passing through the coil to produce a magnetic field. This coil geometry was chosen to have an electrode with a more expanded surface and a more uniform electric field in the medium. During the test, the cells were placed in the culture medium in close proximity to the electrodes. It is important to note that the cells were specifically in the growth medium during the exposure time. There were no modifications to the medium. Cells had complete medium (Fig. 1b). The electric field’s distribution and temperature in the petri dish were also simulated using Comsol 6.0 Multiphysics software. The simulation results showed that a voltage of 0.75V/cm resulted in approximately 63.38% of the petri volume being exposed to a field intensity of (0.33, 0.92] V/cm (Fig. 1c, d). The maximum temperature in the petri dish was found to be 37.13 ˚C (Fig. 1e) based on the simulations, and no significant temperature change was observed experimentally using a thermometer. These findings suggest that the experimental setup provided a controlled and safe means of applying the electric field to the cells.

### Growth curve and determination of doubling time

We constructed a growth curve to determine the different phases of cell development. This curve helps us identify when cells enter the lag phase, the log phase, and the plateau phase. One valuable piece of information we can extract from the growth curve is the doubling time, which refers to the duration it takes for the cell population to double in number.

To calculate the doubling time in a cell curve, we need to have data on the growth rate of the cells. The doubling time refers to the time it takes for the number of cells to double. Here’s a step-by-step guide to calculating the doubling time: First, collect data on the number of cells at different time points during the growth period. Plot this data on a graph to visualize the growth curve. Identify the exponential growth phase, which is characterized by a steep slope on the graph. Calculate the growth rate by determining the slope of the exponential growth phase using linear regression (which showed by red line).

Then, we used the formula Doubling Time.

1 $$\mathrm{N }= {\mathrm{N}}_{0}{\mathrm{e}}^{\mathrm{Bt}}$$

- N represents the final population or quantity at time t.
- N0 represents the initial population or quantity at time t = 0.
- e is the mathematical constant approximately equal to 2.71828.
- B represents the growth rate or the rate of change of the population or quantity over time.

t represents the time elapsed.

U937 and Fs-204 cells were cultured at a density of 1 × 10^5^ cells/ml in 98 wells plates. Then cells were counted every day for 8 days. Logarithmic phase and doubling time were calculated from Eq. 1.

### MTT assay

U937 cells were cultured in 96 wells plates, with different concentrations of DNR (0.001–100 µM) for 4, 24, and 48-h durations. Then a 0.25 mg/ml MTT solution was added to each well after medium exchange and then incubated at 37 ˚C for 3 h protected from light. Consequently, 300 µl DMSO was added to each well to solubilize the purple formazan crystals afterward. The absorbance was then measured by using an Elisa reader spectrophotometer (BioTek, USA) at 570 and 630 nm.

### Electric field and daunorubicin treatments

Experimental groups included: a Sham exposure group, called the control group in the following (The cells received no treatment while there were electrodes in the sham cell petri-dish; they only lacked electric field treatment and DNR); an electric field exposed group (cells exposed solely to the electric field with an average 0.75 V/cm intensity and 200 kHz frequency for 48 h time duration); a DNR treated group (cells treated only with DNR with a concentration of 0.01µM for 48 h time duration), and an electric field + DNR group (cells receiving combination treatment of the electric field of 0.75 V/cm and frequency of 200 kHz with 0.01 µM of DNR for 48 h time duration). The cells were simultaneously exposed to electric field and drug during incubation for 48 h, and the drug remained in the plate throughout the application of electric field. After turning off the application of the electric field, we also removed the drug from the growth medium. Also exposures to all variants (control, electric field alone, DNR, DNR+TTF) were done in parallel. All experiments were repeated three times.

### Membrane permeability analysis and cells number analysis

In this experiment, U937 and Fs-204 cells were cultured in a glass petri dish for 48 h with a density of 100,000 cells/ml, and the cells were treated during the exponentially growing phase. The cells were counted immediately after the end of the treatment time and not incubated. To assess the membrane permeability of the cells after treatment, we utilized the trypan blue dye exclusion method. Viable cells with healthy membranes did not absorb the dye, resulting in a clear and non-stained cytoplasm observed under a microscope. In contrast, cells with affected membranes or that had died showed a blue-stained cytoplasm [26, 27]. To count the cells, we used Methyl green as a cationic stain, which is similar to Ethyl green, commonly used for staining DNA via a hemocytometer.

### Cellular uptake

U937 cells were cultured into a glass petri dish with a density of 100,000 cells/ml and allowed to grow for 48 h. The cells were then treated with DNR in the presence or absence of an electric field and incubated for 24 h. After being washed with cold PBS, the intracellular DNR fluorescent intensity was measured using a CyFlow Space flow cytometer (Partec, Germany), and the emission was collected at 580 nm [28] specifically for DNR. The excitation wavelength used for the DNR measurement was 480 nm, as it is within the typical excitation range for it [1]. The resulting data were analyzed using Flowjo software (version 7.6.1). This experimental setup allowed for the assessment of DNR accumulation in U937 cells in the presence or absence of an electric field, providing insight into the impact of the electric field on drug uptake by the cells.

### Soft agar colony formation assay

The clonogenic assay, also known as the colony formation assay, is an in vitro cell survival assay that evaluates the ability of a single cell to form a colony. A colony is defined as consisting of at least 50 cells and is indicative of a cell’s ability to undergo unlimited division. In this experiment, U937 cells were seeded in a glass petri dish with a density of 100,000 cells/ml and treated during the exponentially growing phase for 48 h. The cells were then centrifuged, and the supernatants were removed then being cultured in a 0.3% agar medium and incubated for colony formation at 37 ˚C in a humidified 5% CO2 atmosphere for 12 days. The resulting colonies were counted, and the plating efficiency (PE) and survival fraction (SF) were determined by the following equations:

2 $$\mathrm{PE }= \frac{\mathrm{Number\, of\, colonies}}{Number\, of\, cells\, seeded} \times 100$$

3 $$\mathrm{SF }= \frac{Number\, of\, colonies }{\mathrm{ Number\, of\, cells\, seeded}\times (\frac{\mathrm{PE}}{100})}$$

### Apoptosis assay

Dual fluorescent staining (AO/EB) acridine orange/ethidium bromide was visualized under a fluorescent microscope [29, 30]. U937 cells were treated after the exponential growth phase for 48 h, and the medium was then replaced with fresh medium. The cells were stained with a solution containing a mixture of EB (100 μg/ml) and AO (100 μg/ml), after which they were imaged using a fluorescent microscope (Zeiss, Germany). To ensure statistical significance, at least 190 to 500 cells were analyzed for each group. This approach allowed for the visualization of any changes in cell morphology or viability resulting from the treatment, providing insight into the treatment’s impact on the cells.

### Comet assay

Alkaline single-cell gel electrophoresis (comet assay) is used to study DNA damages including single-strand breaks (SSBs) and double-strand breaks (DSBs). Upon completion of the 48-h treatment, the cells from each of the 4 groups, were collected and analyzed. About 40,000 cells were suspended in a 0.5% low-melting-point agarose (final concentration) in PBS (pH 7.4). The cells were then suspended by pipette onto a microscopic slide (covered by a thin layer of 1% agarose). After placing agarose in the refrigerator for 10 min, the slides were immersed in the lysis buffer (2.5 M NaCl, 100 mM Na2EDTA, 10 mM Tris, NaOH with a pH of 10.0, and 1% TritonX-100) for 1 h at 4 °C. The slides were then put in the denaturation solution (0.3 M NaOH and 1 mM Na2EDTA; pH > 13) for 30 min. Next, the slides were placed in an electrophoresis tank (20 V, 0.9 V/cm, 251 mA) containing the denaturation solution for 30 min, followed by their transfer into a neutralization buffer. Finally, the prepared slides were stained with EB solution (20 μg/mL). A fluorescence microscope (Axioskop 2 plus; Zeiss, Jena, Germany) was used to obtain comet images. We used the Comet Score 2 software (TriTek Corp, USA) to analyze Comet images. For each group, 50 to 150 cells were analyzed, allowing for the assessment of DNA damage resulting from the treatment.

### Statistics

All data were the result of three independent experiments. Data were indicated as means ± standard error of mean (SEM). For continuous variables, the means were compared by analysis of two sample assuming unequal and equal variances (student t-test). *P* value of 0.05 was considered as the level of statistical significance.

## Results

### Analyzing the impacts of temperature change

In an experimental setting, a Mercury Thermometer was utilized to measure temperature. It was observed that there was no discernible variation in temperature during the electric field exposure time. This observation was further validated by comparing it with the temperature data obtained through temperature simulation using COMSOL modeling. According to the simulations, the maximum temperature recorded in the petri dish was 37.13 ˚C (Fig. 1e). However, when the experiment was conducted using a thermometer, no significant change in temperature was observed. These results indicate that the experimental setup provided a controlled and secure method of applying the electric field to the cells.

### Inferring time-dependent population growth rates in cell cultures to determine doubling times

**Fig. 2:**
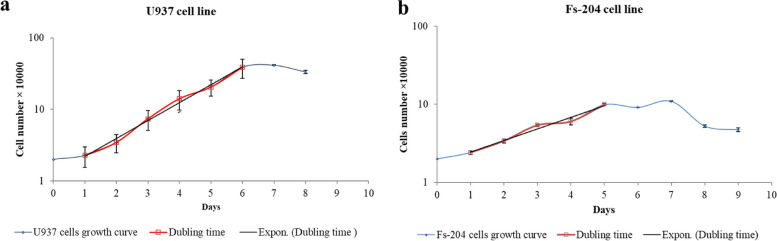
Growth curve and cell number; **a** Growth curve of U937 cells, **b** Growth curve of Fs-204 cells

The growth curve of U937 and Fs-204 cells was obtained by counting the cells daily for 8 days. The doubling times of U937 and Fs-204 cells were calculated during the logarithmic phase, and were found to be 28.82 ± 0.29 and 48.77 ± 3.70 h, respectively (Fig. 2a, b). These results indicate that the growth rate of the Fs-204 cell line was slower than that of U937 cells. Subsequently, cells in the logarithmic growth phase were utilized for further experimentation.

### U937 cell proliferation inhibition by DNR

**Fig. 3:**
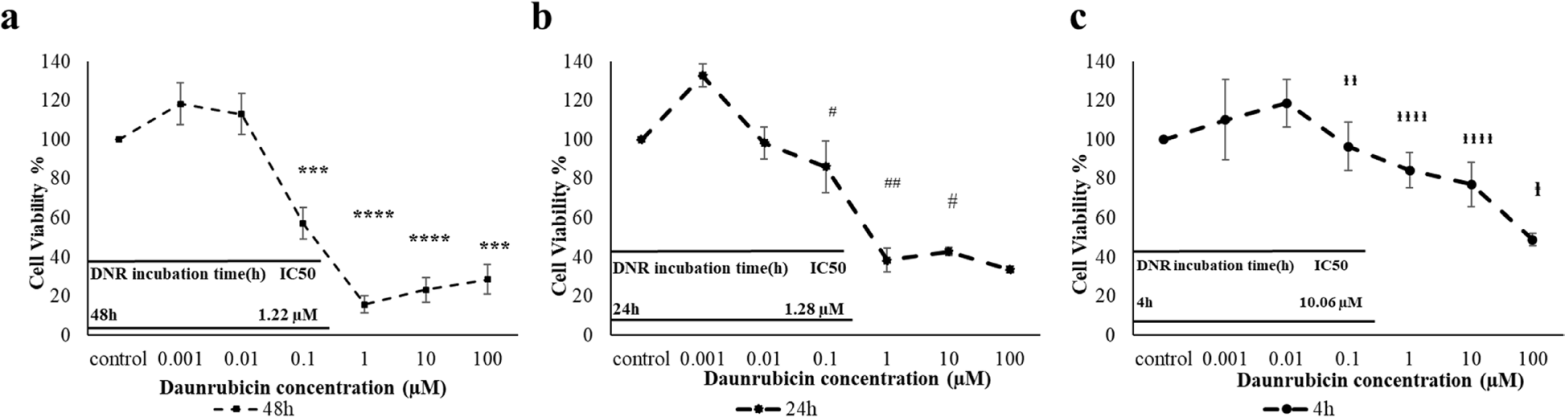
The effect of DNR on the viability of U937 Cells. In this experiment, U937 cells were exposed to various concentrations of DNR ranging from 0.001 to 100 µM for three different durations: **a** 48 h, **b** 24 h, and **c** 4 h. Then the viability of cells was measured by the MTT assay. **** and *** represent *p* ≤ 0.001 and *p* ≤ 0.01, respectively in 48 h, DNR incubation time and in comparison to the control group. ## and # represent *p* ≤ 0.05 and *p* ≤ 0.1 with a 24 h DNR incubation time and comparison to the 48 h incubation. Also,  ,  and  represent *p* ≤ 0.001, *p* ≤ 0.05 and p ≤ 0.1, respectively, with 4 h DNR incubation and comparison to the 48 h incubation

MTT assay was used to evaluate the cytotoxicity of DNR (0.001–100 µM, in three different incubation times: 4, 24, and 48 h) on U937 cells. The results, presented in (Fig. 3), demonstrate that cell survival decreased in a concentration- and time-dependent manner following DNR treatment. Furthermore, the IC50 values obtained from concentration-response curves indicated that the IC50 value decreased with increasing DNR exposure time. Based on these findings, a concentration of 0.01 µM DNR was selected for the 48-h treatment duration to proceed with further testing. IC50(Half maximal inhibitory concentration) is the concentration of an inhibitor where the response is reduced by half. In this case, the vertical axis represents the percentage of cell viability, while the horizontal axis represents the concentration of the drug. We utilized Excel software to plot the data and then used Prism software to determine the IC50 value. Our objective was to identify the dosage that inhibited cell growth by 50%. We insert the final IC50 values for each time point (4, 24, and 48 h) on the graph. Also according to the growth curve of the cells indicates that they are not lacking nutrients until day 6, as they are in the logarithmic phase. The observed changes in the graphs are likely due to the toxicity of the drug, which is more pronounced at the 48-h mark compared to 4 and 24 h.

### Electric field enhanced membrane permeability and inhibited cell growth

**Fig. 4:**
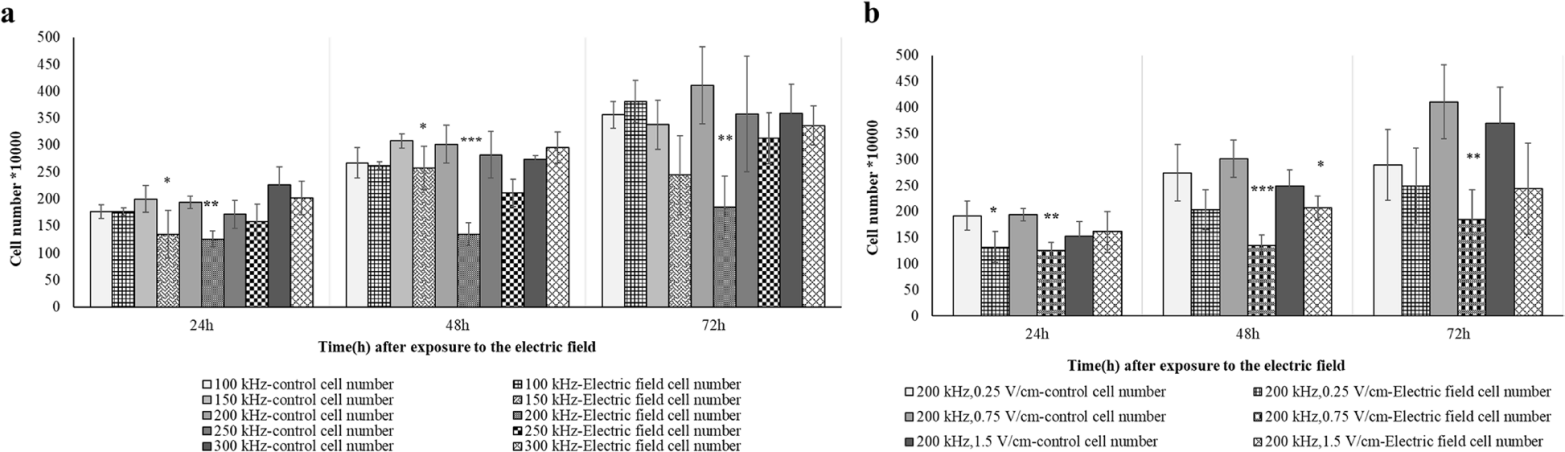
U937 Cell numbers exposed to the electric field. **a** U937 Cell numbers exposed to 100–300 kHz frequency with the duration of 24, 48 and 72 h. *, **, and *** represent *p* ≤ 0.1, *p* ≤ .05, and *p* ≤ 0.01, respectively in U937 cells that were exposed to the electric field compared to the control. **b** U937 Cell numbers in 0.25–1.5 V/cm intensity and duration of 24, 48, and 72 h after exposure to the electric field. *, **, and *** represent *p* ≤ 0.1, *p* ≤ 0.05, and *p* ≤ 0.01, respectively of U937 cells that were exposed to the electric field compared to the control

**Fig. 5:**
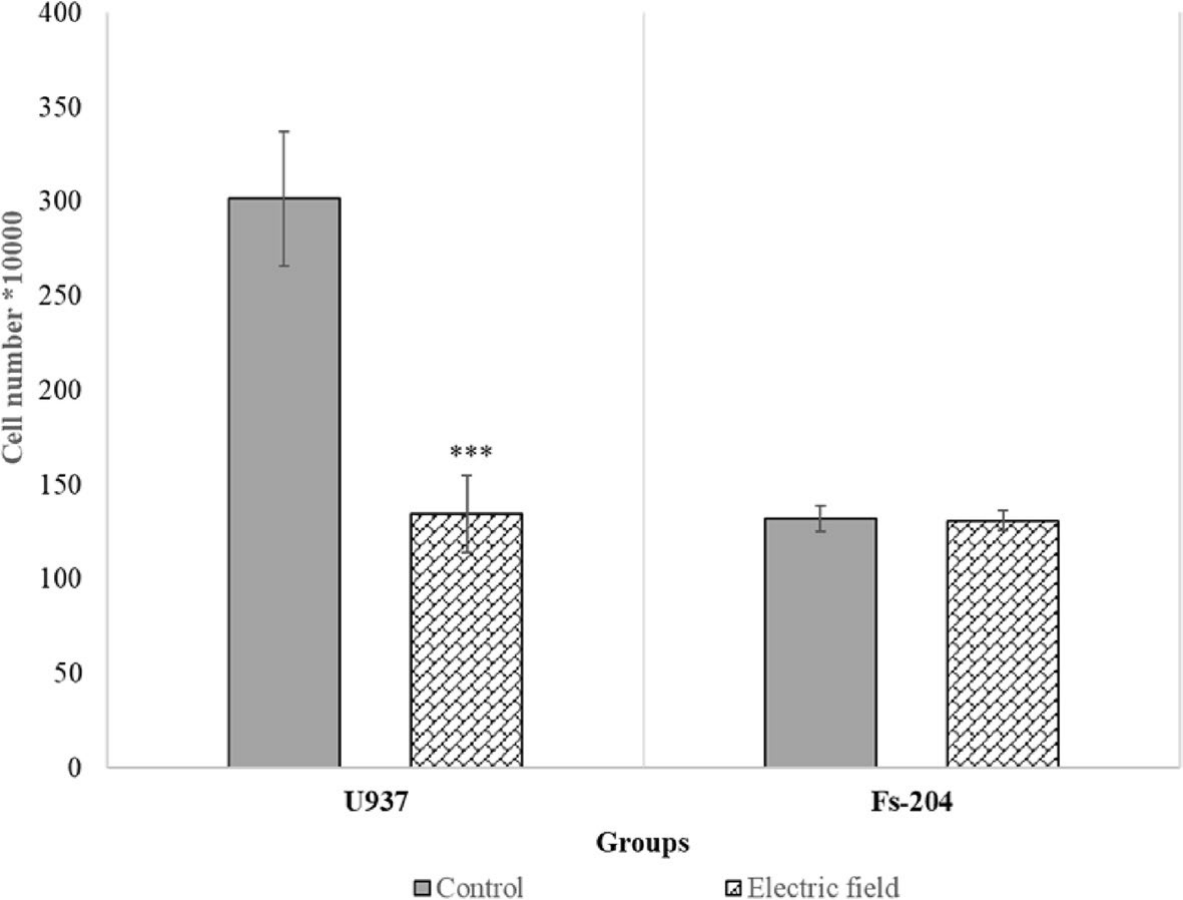
Cell number; Cell numbers (U937 and Fs-204) after 48 h of exposure to the electric field of 0.75 V/cm intensity and 200 kHz frequency. There is a significant difference in U937 cells in comparison with the control, while there was no significant difference in Fs-204 cells in comparison to the control group (*p* ≤ 0.01)

**Fig. 6:**
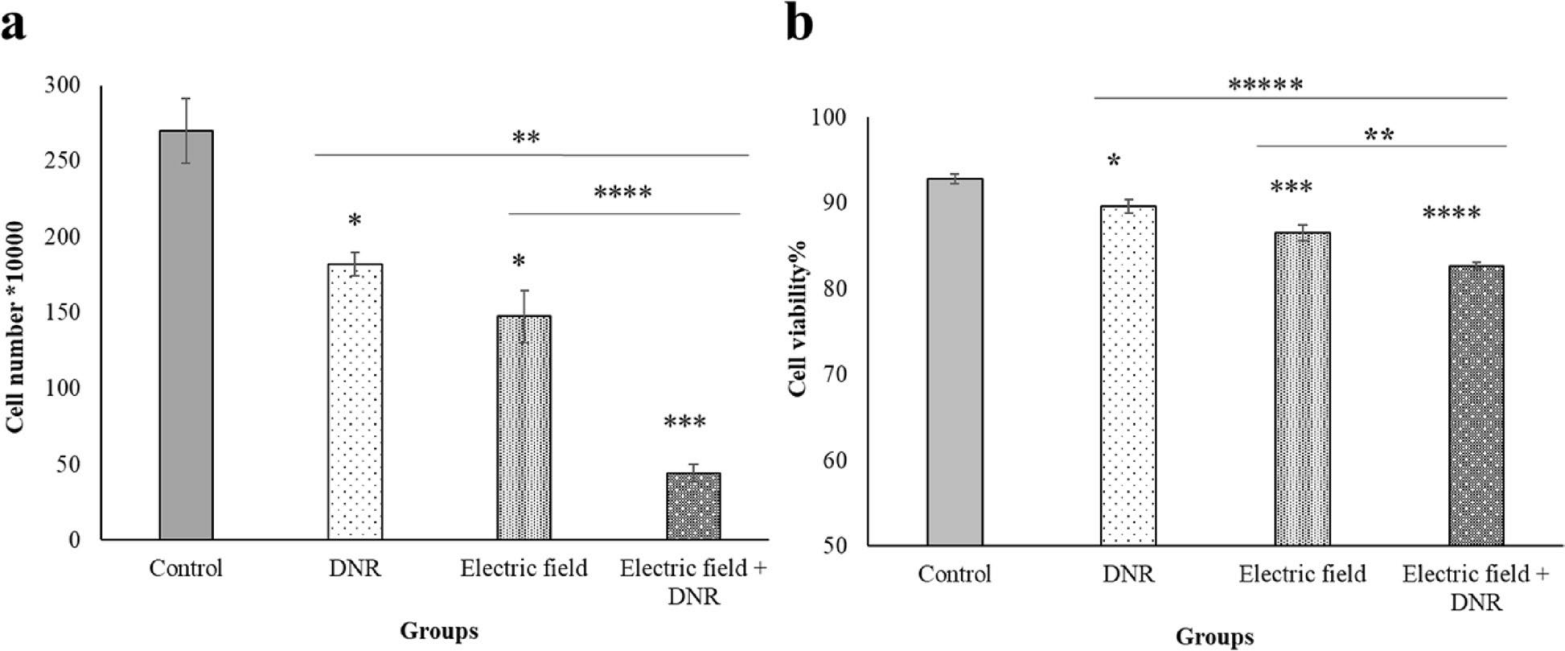
Effect of electric field and DNR on cell numbers and cell viability. **a** Cells numbers of U937 cells exposed to the electric field of 0.75 V/cm intensity and 200 kHz frequency, incubating by DNR 0.01 µM and a combination of the electric field with DNR; were counted by hemocytometer. * and *** represent *p* ≤ 0.01 and *p* ≤ 0.0005, respectively in U937 cells that were incubated by electric field and DNR alone and combination of the electric field with DNR in comparison to the control. Besides ** and **** represent *p* ≤ 0.004 and 0.0001 in cells that incubated by electric field and DNR in comparison to the combination of the electric field with DNR, respectively. **b** Cell viability of U937 cells membrane permeability to trypan blue, after 48 h of electric field exposure with 0.75 V/cm intensity and 200 kHz frequency, DNR 0.01 µM incubation and combination with electric field and DNR were measured by a hemocytometer. *, *** and **** represent *p* ≤ 0.1, *p* ≤ 0.01, and *p* ≤ 0.001, respectively in U937 cells that were incubated by electric field and DNR alone and combination of the electric field with DNR in comparison to the control. Besides ***** represent *p* ≤ 0.0006 in cells that were incubated by DNR in comparison to the combination of the electric field with DNR. Also, ** represents *p* ≤ 0.05 in cells that were exposed by the electric field in comparison to the combination of the electric field with DNR

Cell counting was performed via staining with methyl green and trypan blue. The most significant decrease in cell proliferation was observed at frequency of 200 kHz and intensity of 0.75 V/cm after a 48-h treatment duration (Fig. 4). Furthermore, Fs-204 cells, as normal cells, were exposed to an electric field with an intensity of 0.75 V/cm and a frequency of 200 kHz for 48 h. The statistical analysis revealed no significant changes in the number of cells compared to the control group (Fig. 5). Moreover, there were no notable changes in the cell membrane permeability of the treated cells compared to the control group. The combined treatment of electric field (0.75 V/cm intensity and 200 kHz frequency) and DNR (0.01 µM) showed the highest efficiency in decreasing U937 cells growth (Fig. 6a). These results signify a considerable change in the viability and permeability of U937 cells, as evidenced by alterations in cell membrane permeability to trypan blue dye in the group that received the combined treatment of electric field and DNR (Fig. 6b).

### Drug uptake increased by electric field exposure

**Fig. 7:**
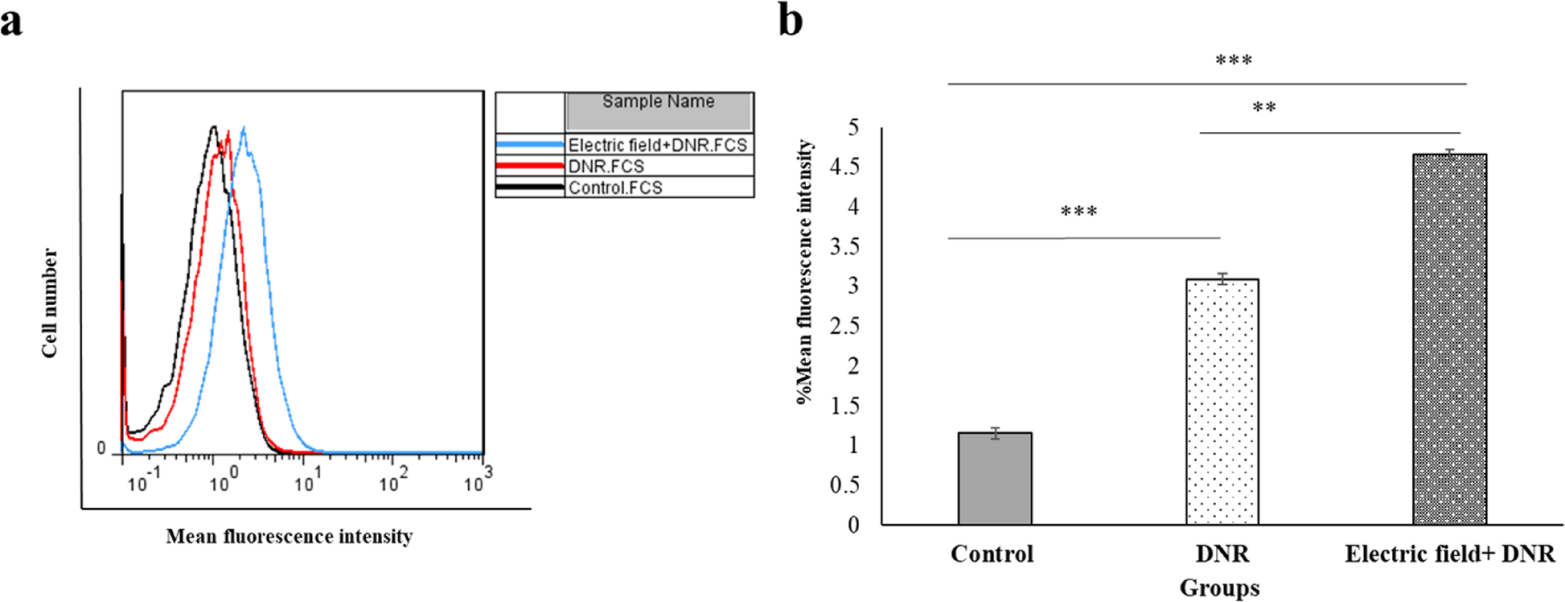
Drug uptake of U937 cells. **a** Flow cytometry was utilized to figure out the intracellular uptake of DNR in U937 cells with the electric field treatment. DNR uptake (0.01 µM), after 24 h of treatment alone and in combination with electric field with 0.75 V/cm intensity and 200 kHz frequency, were studied. Black, red and blue lines respectively; represent control, DNR, and electric field + DNR groups. **b** Intracellular DNR uptake in U937 cells in the presence and absence of the electric field. The mean fluorescence intensity of the U937 cells was increased while exposed to the electric field. ** and *** represent *p* ≤ 0.05 and *p* ≤ 0.001, respectively

To investigate the effect of the electric field on increasing DNR uptake and enhancing membrane permeability, the accumulation of DNR in U937 cells was measured using flow cytometry. As illustrated in (Fig. 7), the average fluorescence intensity in the DNR + Electric field group was significantly higher compared to that of the DNR and free DNR groups. These results suggest that the electric field facilitated greater penetration of DNR into the cells during the 24-h incubation period.

### Inhibited colony formation using only electric field exposure and in combination with DNR treatment

**Fig. 8:**
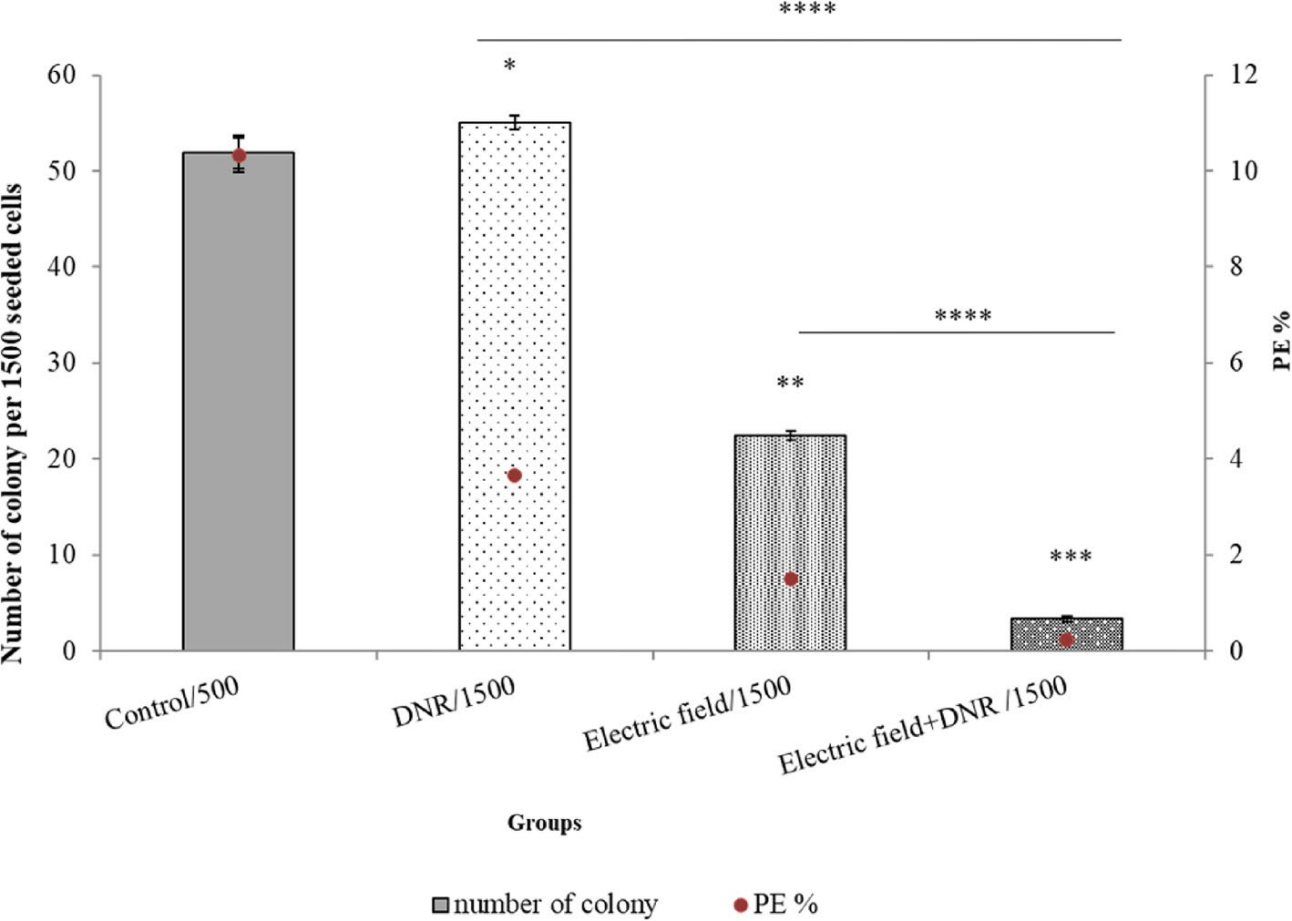
U937 clonogenic survival assay. The colony number of control was 51.9 out of 500 cells-cultured. While colony number of electric field and DNR treatment and combination of the electric field with DNR treatment were 55, 22.4, and 3.3 colonies out of 1500 cell-cultured, respectively. Plating efficiency (PE) of DNR (0.01 µM) and the electric field (200 kHz, 0.75 V/cm) alone and in combination had a significant difference in comparison to control. *, ** and ***, *p* ≤ 0.005, *p* ≤ 0.001 and *p* ≤ 0.0008 from control groups, respectively. Also, **** represents *p* ≤ 0.00001, in comparison to DNR + electric field by DNR and electric field groups

To evaluate the effects of the electric field (with a frequency of 200 kHz and intensity of 0.75 V/cm) and DNR (0.01 μM) on U937 cells, a soft agar assay was conducted either alone or in combination with each other for 48 h. After 12 days of incubation, untreated U937 cells displayed a clonogenic efficiency of 10.3%. In contrast, cells treated solely with the electric field or DNR demonstrated reduced efficiencies of 3.6% and 1.4%, respectively. Furthermore, the combination of the electric field with DNR exhibited the most significant decrease in clonogenic efficiency, with a value of 0.22% (Fig. 8).

### Electric field exposure alone and in combination with DNR, increased cell death

**Fig. 9:**
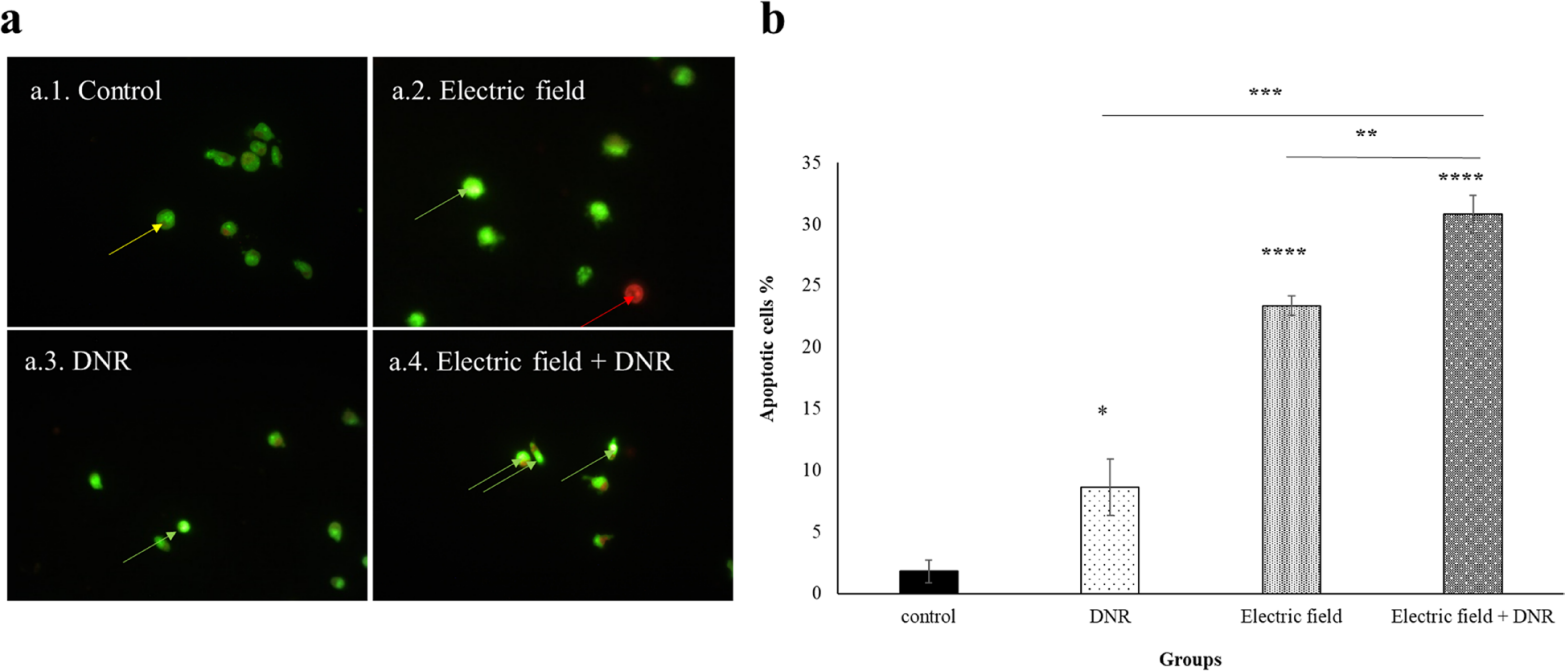
Fluorescence micrographs of U937 cells obtained by fluorescence microscopy. **a** In these micrographs. a.1. Control group; a.2. Electric field group; a.3. DNR group; a.4. Electric field + DNR group, viable, early apoptotic, and late apoptotic cells are shown by yellow, green, and red arrows, respectively. **b** Apoptotic death significantly increased in the electric field exposed group (200 kHz, 0.75 V/cm) in comparison with the control group. Combined treatment of DNR and electric field indicates increased levels of apoptotic death. * and ***** represent *p* ≤ 0.05, *p* ≤ 0.0001, respectively. Also, *** and ** represent *p* ≤ 0.001 and *p* ≤ 0.01 in cells that were incubated by DNR and electric field in comparison to the combination of the electric field with DNR, respectively

The apoptotic effects of the electric field (with a frequency of 200 kHz and intensity of 0.75 V/cm) alone or in combination with DNR (0.01 μM) on U937 cells were investigated for a period of 48 h using AO/Et dual staining. Apoptotic cells were identified by dense chromatin and fragmented nuclei, while living cells showed well-distributed chromatin. The results depicted in (Fig. 9) showed a significant increase in apoptotic death in the group exposed to the electric field. Moreover, the combination treatment group demonstrated a significant increase in apoptotic mortality compared to the control group.

### The effect of electric field alone and in combination with DNR on cell function (DNA damage and repair)

**Fig. 10:**
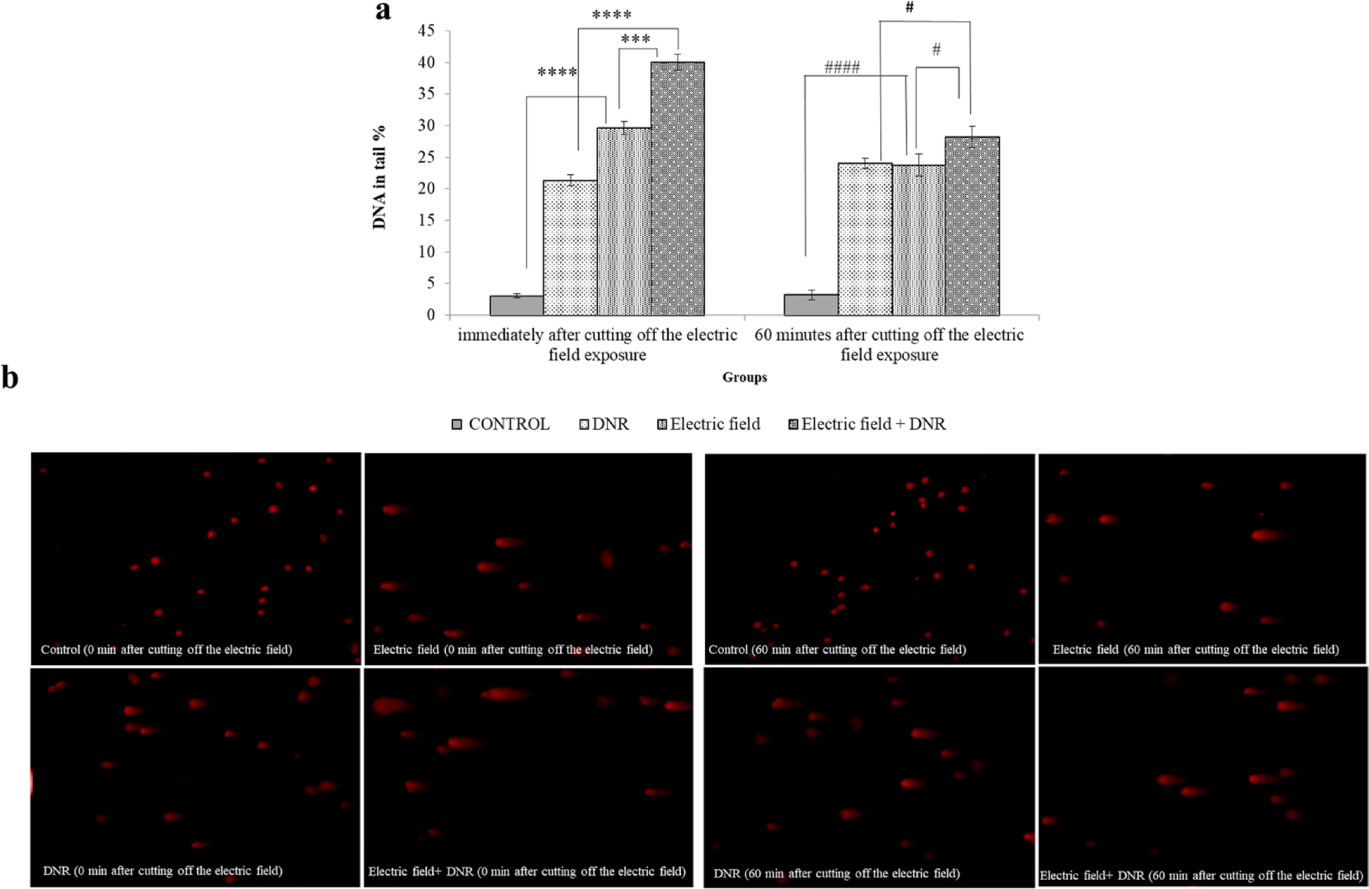
DNA damages. **a** DNA damages increased significantly immediately after 48 h treatment with the electric field (200 kHz, 0.75 V/cm) alone and in combination with DNR in comparison to control. **** and ***, represent *p* ≤ 0.001, *p* ≤ 0.01 directly after stopping electric field exposure, respectively. Also, DNA repair was shown after 60 min of stopping exposed cells to the electric field alone and in the cells that were in the combination treatment. #### and # represent *p* ≤ 0.001 and *p* ≤ 0.1 in 60 min after stopping electric field exposure, respectively. **b** Represent comet images at immediately after cutting off the electric field and 60 min after cutting off electric field exposure in all four groups: control, DNR, electric field, and electric field + DNR groups

Using the comet assay, we evaluated the extent of DNA damage by measuring the DNA in the tail (%) for the electric field, DNR treatment alone, and in combination with each other compared to the control group, both at 0 and 60 min after intercepting the electric field (Fig. 10). Our findings indicated that exposure to the electric field and treatment with DNR separately resulted in a significant level of DNA damage. Interestingly, after turning off the electric field for 60 min, the extent of DNA damage was significantly repaired.

## Discussion

As commenced our investigation, there was a lack of reported data regarding the response of non-adherent cells to electric field treatment. Therefore, our objective was to investigate whether blood cells (specifically U937 cells) with a spherical structure and suspended in a growth medium could be effectively treated with electric fields. This research is particularly significant in the context of leukemia, as traditional cancer treatments often have severe side effects and can harm healthy cells in the surrounding tissue. Utilizing targeted therapy like TTF, which specifically acts on cancer cells while sparing healthy cells, provides a more favorable treatment option for leukemia patients who may already be experiencing significant side effects. Since TTF can be used locally in body, we propose electric field exposure to target the tumor cells that are located in the bone marrow, spleen, or lymph nodes. Noting that cells of tissues studied in vitro and in vivo are adherent, their cell shapes are much different from non-adherent spherical cells. The calculated electric field intensity distributions, in the irregular cell, are much more non-uniform than that in spherical cells [12]; this non-uniformity can relatively increase phenomena like dielectrophoresis. Since blood cells are mainly observed as spherical, it is important to specifically study blood cells, as suspended spherical cells when exposed to electric field with intermediate frequencies and low intensities. The wide variety of tumors and cell characteristics might be affected by electric field as a physical treatment modality [23] doubling time is one of the most dominant factors that come into view [3, 21]. As stated in the literatures, electric fields selectively could interfere with fast-growing cells but not slow-growing cells [31, 32]. According to our results, the doubling time of U937 cells is 1.7 folded of the Fs-204 (Fig. 2a, b). Our results further indicate that while electric fields do not significantly impact the proliferation and membrane permeability of Fs-204 cells - a normal cell line in suspension - they do affect U937 cells (Fig. 5).

We conducted a study to investigate the feasibility of using a non-invasive treatment approach for a leukemia cell line, utilizing an electric field with a frequency of 200 kHz and an intensity of 0.75 V/cm, in combination with DNR. The proposed mechanism of action for DNR is to disrupt the synthesis of macromolecules by interacting with DNA double strands, as outlined in reference [33]. Our results indicate that both the electric field alone and in combination with DNR at a concentration of 0.01 µM led to a significant reduction in the proliferation of the U937 cell line (Fig. 6a). These findings suggest that the electric field could be a promising therapeutic option for leukemia cells, either as a standalone treatment or in combination with DNR.

According to the existing literature, a novel mechanism of action for the electric field is its ability to increase cancer cell membrane permeability, as noted in reference [34]. Notably, previous experimental results have demonstrated a difference in membrane potential between cancer cells and normal cells, suggesting that TTFields may have a more pronounced effect on the membrane potential of dividing cells, while having a negligible impact on normal cells [10]. Moreover, since tumor cells possess a higher membrane permeability than normal cells [35], their membrane conductivity should also be higher [10]. Additionally, the electric field has been found to alter the cellular membrane structure, but did not induce membrane permeability in normal human fibroblasts, suggesting that this phenomenon may be specific to cancer cells [36].

In line with the previous results for the adherent cells, our results showed that the U937 cell membrane became more permeable to trypan blue after electric field exposure (200 kHz and 0.75 V/cm) (Fig. 6b). Moreover, we showed that the electric field facilitated the penetration of more DNR into U937 cells, leading to a reduction in cell proliferation. The marked increase in drug uptake observed in the combination of DNR with the electric field group, compared to the DNR and free DNR groups (Fig. 7), provides further evidence of the electric field’s significant effect on cell membrane permeability, which can cause considerable death of U937 cancer cells. Since we utilized the MTT test to assess the level of drug toxicity, specifically focusing on determining the appropriate dosage of DNR. Our objective was to identify a dose that would not have a toxic impact on the cells independently. Consequently, we employed a concentration of 0.01 µM DNR in combination with an electric field to examine the effect of the electric field in enhancing the drug’s efficacy. Subsequently, we used the colony assay to evaluate the cell’s capacity to reproduce following exposure to both the drug and the electric field. Our observations revealed a decrease in colonization when the DNR was combined with the electric field, as opposed to when either the DNR or the electric field was administered alone (Fig. 8). These results are consistent with previous findings for adherent cells, as reported in reference [37]. Further investigations of adherent cells have demonstrated that the electric field can increase apoptotic death when cells are exposed to the electric field alone or in combination with a chemotherapeutic agent, as outlined in references [15, 38]. Our data also reveal that exposure to the electric field alone, as well as in combination with DNR, led to a significant increase in apoptosis in the U937 cell line (Fig. 9).

Our findings are consistent with other studies that have used comet analysis to demonstrate an immediate increase in DNA damage following exposure to the electric field, as noted in reference [4]. In this group, DNA damage was observed to be repaired within 60 min after exposure to the electric field. Our results further indicate that a combination of the electric field and DNR led to an increase in DNA damage (Fig. 10). We hypothesize that by increasing cell membrane permeability, exposure to the electric field facilitated the penetration of more drugs into U937 cells, resulting in increased DNA damage. Similarly, after disconnecting the electric field in the presence of DNR for 60 min, we observed a significant reduction in the rate of DNA damage, possibly due to a decrease in cell membrane permeability. This may have resulted in less drug penetrating the cells and allowed for DNA damage to be repaired.

Based on our observed results, the electric field affected the spherical suspended cells. The electric field with the 0.75 V/cm intensity and frequency 200 kHz for 48 h time duration, has effects on U937 cells, while having no discernible effect on FS-204, a normal B lymphoblastoid cell line. Furthermore, our findings suggest that the combination of electric field therapy with DNR enhances the efficacy of electric field therapy in leukemia cells (U937). Despite these promising results, the mechanism by which the electric field affects cancer cells remain unknown. However, our data suggest that electric field therapy leads to an increase in cell membrane permeability. Given that the electric field has been shown to affect cell membrane potential, future studies could be conducted to investigate the role of ion channels in this process.

This study, aimed to investigate the effect of the electric field on blood cancer cells, which have a spherical structure and are suspended. Previous theoretical calculations suggested that the electric field distribution is uniform in spherical models, but locally stronger in structured cells which have edges and special shapes [12]. Also, all previous laboratory tests were conducted on adherent cells or solid tumors [19], which have different shape structures. We found that the electric field can influence non-adherent and spherical cells, regardless of their structure and shape. Also discovered that the electric field can enhance the effectiveness of chemotherapy on U937 cells.

Since These findings show that the electric field can be a promising treatment option for leukemia cells as an independent treatment or in combination with DNR, it seems that it can be a suitable treatment method for patients with leukemia cancer. So there must be designed some electrodes suitable for patients with leukemia and the exact placement and configuration of the electrodes may vary depending on the specific treatment protocol and the location of the leukemia cells. We suggest using electrodes to deliver electrical currents to the body, which can target the tumor cells that are located in the bone marrow, spleen, or lymph nodes. By applying electrodes on the skin near these places, we can expose the tumor cells to tumor-treating fields (TTF), which can disrupt their cell division and induce cell death. In order to ascertain the positive impact of the electric field on leukemia, it is imperative to conduct experiments on animals initially, followed by subsequent trials involving human subjects.

## Data Availability

All data generated or analysed during this study are included in this published article.
